# Crystal structure of 3-amino-1-(4-meth­oxy­phen­yl)-1*H*-benzo[*f*]chromene-2-carbo­nitrile

**DOI:** 10.1107/S2056989015011020

**Published:** 2015-06-13

**Authors:** Shaaban K. Mohamed, Peter N. Horton, Mehmet Akkurt, Sabry H. H. Younes, Mustafa R. Albayati

**Affiliations:** a Chemistry and Environmental Division, Manchester Metropolitan University, Manchester M1 5GD, England; b Chemistry Department, Faculty of Science, Minia University, 61519 El-Minia, Egypt; c School of Chemistry, University of Southampton, Highfield, Southampton, SO17 1BJ, England; d Department of Physics, Faculty of Sciences, Erciyes University, 38039 Kayseri, Turkey; e Chemistry Department, Faculty of Science, Sohag University, 82524 Sohag, Egypt; f Kirkuk University, College of Science, Department of Chemistry, Kirkuk, Iraq

**Keywords:** crystal structure, chromene compounds, benzochromene, hydrogen bonding, C—H⋯π inter­actions

## Abstract

In the title compound, C_21_H_16_N_2_O_2_, the meth­oxy­benzene ring is almost perpendicular to the mean plane of the naphthalene ring system, making a dihedral angle of 83.62 (5)°. The 4*H*-pyran ring fused with the naphthalene ring system is almost planar [maximum deviation = 0.033 (1) Å]. In the crystal, mol­ecules are linked into inversion dimers by pairs of N—H⋯N hydrogen bonds. N—H⋯O hydrogen bonds connect the dimers, forming a helical supra­molecular chain along the *a*-axis direction. The crystal packing also features C—H⋯π inter­actions.

## Related literature   

For the biological inter­est of benzochromene derivatives, see: Gourdeau *et al.* (2004 ▸); Sangani *et al.* (2012 ▸); Cheng *et al.* (2003 ▸); Kamal *et al.* (2012 ▸); Denish *et al.* (2012 ▸); Nitin *et al.*. (2012 ▸); Bhat *et al.* (2008 ▸). For a similar structure, see: Akkurt *et al.* (2013 ▸).
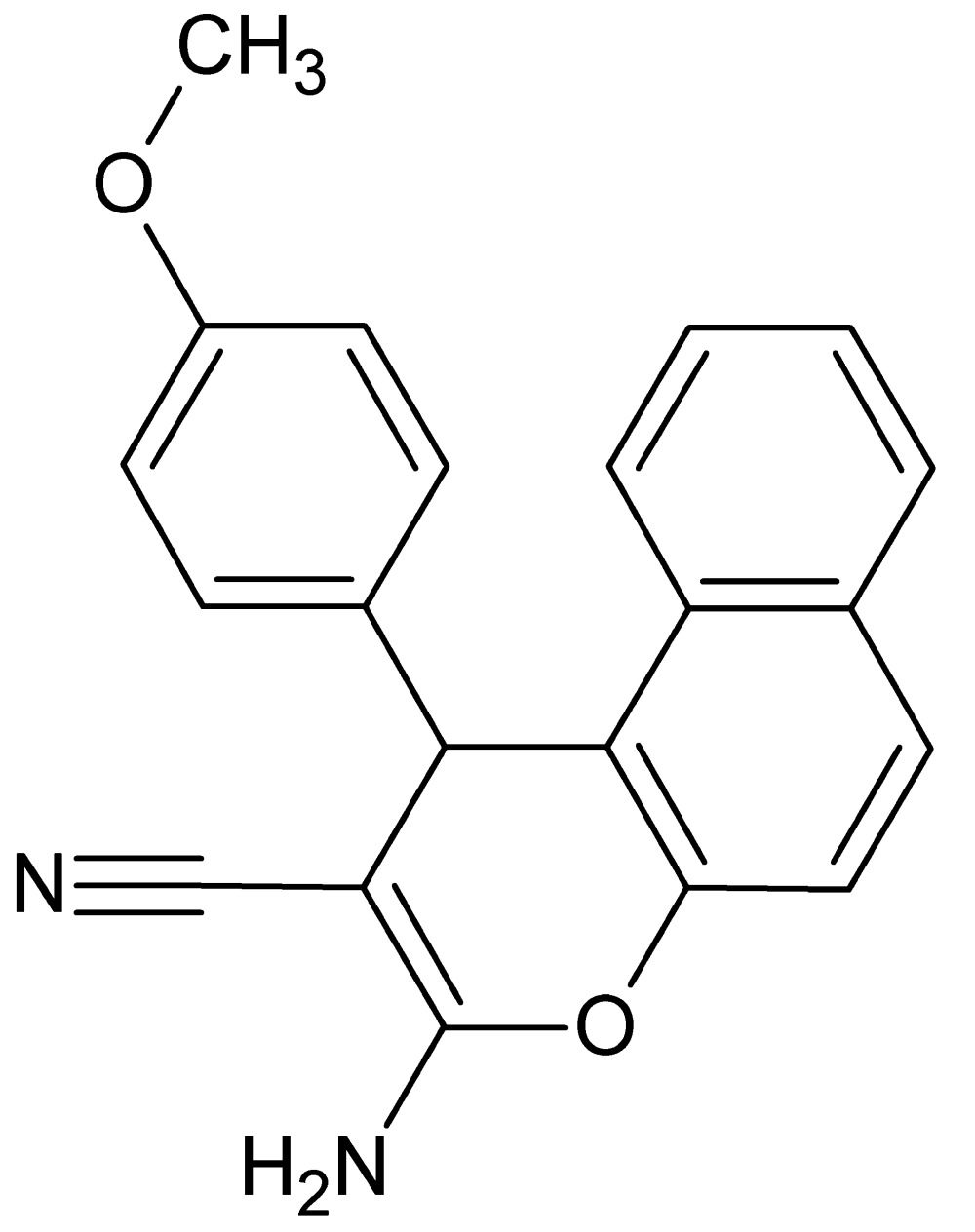


## Experimental   

### Crystal data  

C_21_H_16_N_2_O_2_
*M* *_r_* = 328.36
Monoclinic, 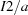

*a* = 20.6017 (14) Å
*b* = 6.1461 (4) Å
*c* = 25.9689 (16) Å
β = 94.332 (4)°
*V* = 3278.8 (4) Å^3^
*Z* = 8
Cu *K*α radiation
μ = 0.70 mm^−1^
*T* = 100 K
0.38 × 0.23 × 0.13 mm

### Data collection  

Rigaku AFC11 diffractometer
Absorption correction: multi-scan (*CrystalClear-SM Expert*; Rigaku, 2012 ▸) *T*_min_ = 0.910, *T*_max_ = 1.000
12941 measured reflections
2914 independent reflections
2832 reflections with *I* > 2σ(*I*)
*R*_int_ = 0.037

### Refinement  

*R*[*F* ^2^ > 2σ(*F* ^2^)] = 0.041
*wR*(*F* ^2^) = 0.116
*S* = 1.03
2914 reflections
236 parameters
H atoms treated by a mixture of independent and constrained refinement
Δρ_max_ = 0.31 e Å^−3^
Δρ_min_ = −0.23 e Å^−3^

### 

Data collection: *CrystalClearSM Expert* (Rigaku, 2012 ▸); cell refinement: *CrystalClearSM Expert*; data reduction: *CrystalClearSM Expert*; program(s) used to solve structure: *SUPERFLIP* (Palatinus & Chapuis, 2007 ▸); program(s) used to refine structure: *SHELXL2014* (Sheldrick, 2015 ▸); molecular graphics: *ORTEP-3 for Windows* (Farrugia, 2012 ▸); software used to prepare material for publication: *WinGX* (Farrugia, 2012 ▸).

## Supplementary Material

Crystal structure: contains datablock(s) global, I. DOI: 10.1107/S2056989015011020/hg5445sup1.cif

Structure factors: contains datablock(s) I. DOI: 10.1107/S2056989015011020/hg5445Isup2.hkl

Supporting information file. DOI: 10.1107/S2056989015011020/hg5445Isup3.cml

**Fig. 1.**
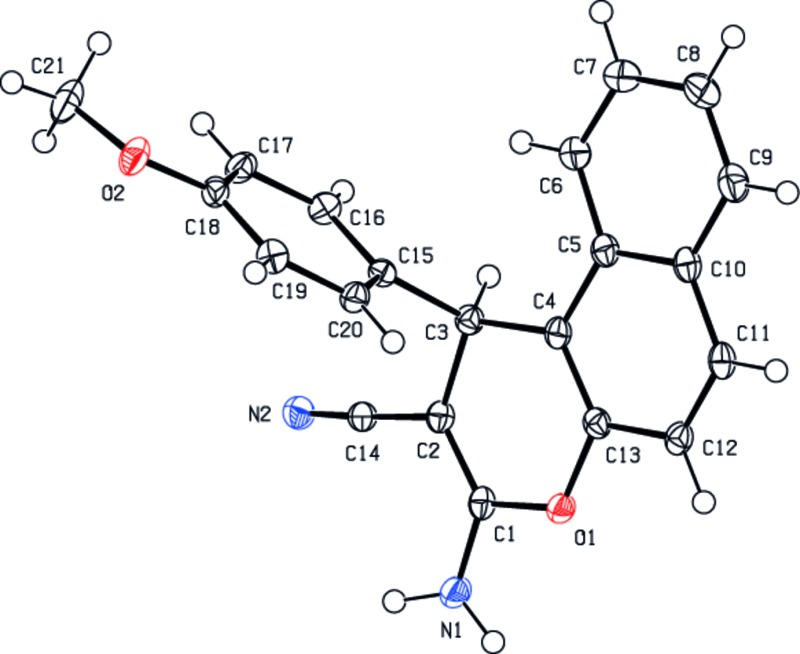
View of the title compound with the atom numbering scheme. Displacement ellipsoids for non-H atoms are drawn at the 50% probability level.

. DOI: 10.1107/S2056989015011020/hg5445fig1.tif
View of the title compound with the atom numbering scheme. Displacement ellipsoids for non-H atoms are drawn at the 50% probability level.

**Fig. 2.**
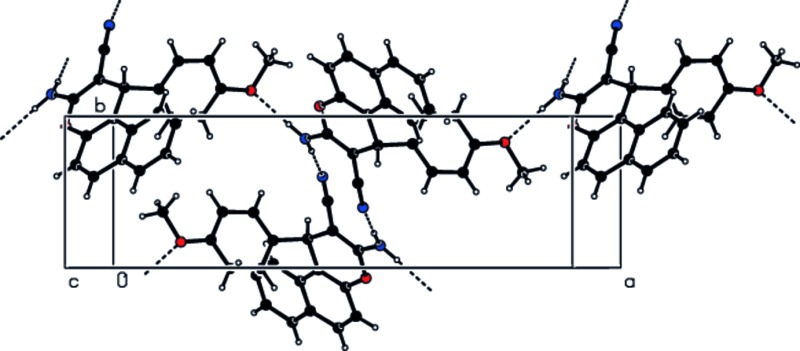
View of the dimers forming by N—H···N hydrogen bonds.

. DOI: 10.1107/S2056989015011020/hg5445fig2.tif
View of the dimers forming by N—H⋯N hydrogen bonds.

CCDC reference: 1405398

Additional supporting information:  crystallographic information; 3D view; checkCIF report

## Figures and Tables

**Table 1:** Hydrogen-bond geometry (, ) *Cg*2 is the centroid of the C4/C5/C10C13 ring.

*D*H*A*	*D*H	H*A*	*D* *A*	*D*H*A*
N1H1*A*N2^i^	0.896(18)	2.125(18)	3.0174(15)	173.8(16)
N1H1*B*O2^ii^	0.900(17)	2.053(17)	2.9509(14)	175.5(14)
C11H11*Cg*2^iii^	0.95	2.56	3.3913(14)	147

Symmetry codes: (i) 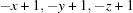; (ii) 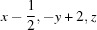; (iii) 
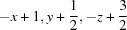
.

## supplementary crystallographic information

### S1. Comment

Benzopyran (Chromene) is one of the important medicinal pharmacophores found in natural compounds which generated great attention because of their interesting biological activity. The natural and synthetic chromene derivatives have important biological activities such as antivascular (Gourdeau *et al.*, 2004), antimicrobial (Sangani *et al.*, 2012), TNF-α inhibitor (Cheng *et al.*, 2003), anticancer (Kamal *et al.*, 2012), anti-HIV (Denish *et al.*, 2012), anti-inflammatory (Nitin *et al*., 2012), and anticonvulsant activity (Bhat *et al.*, 2008). Based on such findings and following to our study on synthesis of bio-active heterocyclic molecules we herein report the synthesis and crystal structure of the title compound.

Fig. 1 shows the asymmetric unit of the title compound. The methoxybenzene ring (C15–C20) is approximately perpendicular to the naphthalene ring system [C4–C13, maximum deviation = 0.040 (1) Å at atom C12] as indicated by the dihedral angle of 83.62 (5)°. The pyran ring (O1/C1–C4/C13) is almost planar [maximum deviation = -0.033 (1) Å at atom C2]. The methoxy group (C21/O2) is nearly co-planar with the attached benzene ring (C15–C20) with the torsion angle C21—O2—C18—C19 of -171.07 (11)°. The bond lengths and angles in the title compound are within normal ranges and comparable with those reported for a similar structure (Akkurt *et al.*, 2013).

In the crystal, molecules are linked into a helical supramolecular chain along the *a* axis, which consist of N1—H1B···O2 hydrogen bonds that connect the dimers formed by N1—H1A···N2 hydrogen bonds, to each other (Fig. 2). The crystal packing is further stabilized by C—H···π interactions (Table 1).

### S2. Experimental

An ethanolic solution of 4-methoxybenzylidenepropanedinitrile (184 mg; 1 mmol) and 2-naphthol (144 mg; 1 mmol) was refluxed with stirring for 3 h at 350 K with adding two drops of piperidine. The solid product was obtained by cooling the reaction mixture to room temperature, then it was collected by filtration, washed with cold ethanol and dried under vacuum. Colourless crystals of the title compound (*M*.p. 465 K) suitable for X-ray diffraction were obtained in excellent yield (87%) by recrystallization of the crude product from ethanol using the slow evaporation method.

### S3. Refinement

All H atoms attached to C atoms were fixed geometrically and treated as riding with C—H = 0.95 Å (aromatic CH), C—H = 0.98 Å (methyl CH_3_), C—H = 1.00 Å (methine CH) with *U*_iso_(H) = 1.5*U*_eq_(methyl C) or *U*_iso_(H) = 1.2*U*_eq_(C). The H atoms of the NH_2_ group were located in difference Fourier maps and included in the subsequent refinement using restraints (N1—H1B = 0.900 (17) Å and N1—H1A = 0.896 (18) Å) with *U*_iso_(H) = 1.2*U*_eq_(N).

### Crystal data

**Table:**

C_21_H_16_N_2_O_2_	*F*(000) = 1376
*M**_r_* = 328.36	*D*_x_ = 1.330 Mg m^−^^3^
Monoclinic, *I*2/*a*	Cu *K*α radiation, λ = 1.54178 Å
Hall symbol: -I 2ya	Cell parameters from 15816 reflections
*a* = 20.6017 (14) Å	θ = 2.6–68.3°
*b* = 6.1461 (4) Å	µ = 0.70 mm^−^^1^
*c* = 25.9689 (16) Å	*T* = 100 K
β = 94.332 (4)°	Prism, colourless
*V* = 3278.8 (4) Å^3^	0.38 × 0.23 × 0.13 mm
*Z* = 8	

### Data collection

**Table:**

Rigaku AFC11 diffractometer	2914 independent reflections
Radiation source: Rotating Anode	2832 reflections with *I* > 2σ(*I*)
Detector resolution: 22.2222 pixels mm^-1^	*R*_int_ = 0.037
profile data from ω–scans	θ_max_ = 68.2°, θ_min_ = 3.4°
Absorption correction: multi-scan (*CrystalClearSM Expert*; Rigaku, 2012)	*h* = −24→24
*T*_min_ = 0.910, *T*_max_ = 1.000	*k* = −5→7
12941 measured reflections	*l* = −31→24

### Refinement

**Table:**

Refinement on *F*^2^	Hydrogen site location: mixed
Least-squares matrix: full	H atoms treated by a mixture of independent and constrained refinement
*R*[*F*^2^ > 2σ(*F*^2^)] = 0.041	*w* = 1/[σ^2^(*F*_o_^2^) + (0.0752*P*)^2^ + 2.3851*P*] where *P* = (*F*_o_^2^ + 2*F*_c_^2^)/3
*wR*(*F*^2^) = 0.116	(Δ/σ)_max_ = 0.001
*S* = 1.03	Δρ_max_ = 0.31 e Å^−^^3^
2914 reflections	Δρ_min_ = −0.23 e Å^−^^3^
236 parameters	Extinction correction: *SHELXL2014* (Sheldrick, 2015), FC^*^=KFC[1+0.001XFC^2^Λ^3^/SIN(2Θ)]^-1/4^
0 restraints	Extinction coefficient: 0.0014 (2)

### Special details

**Table:**

Geometry. Bond distances, angles *etc*. have been calculated using the rounded fractional coordinates. All su's are estimated from the variances of the (full) variance-covariance matrix. The cell e.s.d.'s are taken into account in the estimation of distances, angles and torsion angles
Refinement. Refinement on *F*^2^ for ALL reflections except those flagged by the user for potential systematic errors. Weighted *R*-factors *wR* and all goodnesses of fit *S* are based on *F*^2^, conventional *R*-factors *R* are based on *F*, with *F* set to zero for negative *F*^2^. The observed criterion of *F*^2^ > σ(*F*^2^) is used only for calculating -*R*-factor-obs *etc*. and is not relevant to the choice of reflections for refinement. *R*-factors based on *F*^2^ are statistically about twice as large as those based on *F*, and *R*-factors based on ALL data will be even larger.

### Fractional atomic coordinates and isotropic or equivalent isotropic displacement parameters (Å^2^)

**Table:**

	*x*	*y*	*z*	*U*_iso_*/*U*_eq_	
O1	0.46524 (4)	1.06769 (14)	0.61312 (3)	0.0180 (2)	
O2	0.82346 (4)	0.82919 (15)	0.54600 (3)	0.0243 (3)	
N1	0.43089 (5)	0.85280 (19)	0.54736 (4)	0.0229 (3)	
N2	0.54448 (5)	0.39845 (17)	0.54907 (4)	0.0212 (3)	
C1	0.47727 (6)	0.88455 (19)	0.58585 (5)	0.0171 (3)	
C2	0.52947 (6)	0.75466 (19)	0.59874 (5)	0.0172 (3)	
C3	0.58264 (6)	0.80882 (19)	0.64036 (4)	0.0166 (3)	
C4	0.56459 (6)	1.01315 (19)	0.66861 (4)	0.0166 (3)	
C5	0.60414 (6)	1.0884 (2)	0.71278 (5)	0.0184 (3)	
C6	0.65975 (6)	0.9729 (2)	0.73361 (5)	0.0233 (4)	
C7	0.69636 (7)	1.0479 (3)	0.77625 (5)	0.0303 (4)	
C8	0.68002 (7)	1.2444 (3)	0.80039 (5)	0.0301 (4)	
C9	0.62635 (6)	1.3586 (2)	0.78173 (5)	0.0245 (4)	
C10	0.58687 (6)	1.2843 (2)	0.73818 (5)	0.0190 (3)	
C11	0.52962 (6)	1.39706 (19)	0.71978 (5)	0.0189 (3)	
C12	0.49085 (6)	1.3200 (2)	0.67886 (5)	0.0181 (3)	
C13	0.50912 (6)	1.12794 (19)	0.65376 (4)	0.0164 (3)	
C14	0.53658 (5)	0.5592 (2)	0.57068 (4)	0.0170 (3)	
C15	0.64862 (5)	0.8191 (2)	0.61700 (4)	0.0162 (3)	
C16	0.68716 (6)	0.6343 (2)	0.61775 (5)	0.0192 (3)	
C17	0.74603 (6)	0.6302 (2)	0.59448 (5)	0.0204 (3)	
C18	0.76660 (6)	0.8158 (2)	0.57027 (5)	0.0189 (3)	
C19	0.72851 (6)	1.0035 (2)	0.56930 (5)	0.0198 (3)	
C20	0.66979 (6)	1.0046 (2)	0.59208 (4)	0.0180 (3)	
C21	0.85852 (6)	0.6305 (2)	0.53986 (6)	0.0306 (4)	
H1A	0.4404 (8)	0.772 (3)	0.5202 (7)	0.031 (4)*	
H1B	0.3976 (8)	0.948 (3)	0.5451 (6)	0.028 (4)*	
H3	0.58470	0.68680	0.66590	0.0200*	
H6	0.67180	0.84130	0.71770	0.0280*	
H7	0.73310	0.96680	0.78970	0.0360*	
H8	0.70620	1.29690	0.82940	0.0360*	
H9	0.61530	1.49000	0.79820	0.0290*	
H11	0.51810	1.52820	0.73620	0.0230*	
H12	0.45200	1.39460	0.66740	0.0220*	
H16	0.67320	0.50720	0.63450	0.0230*	
H17	0.77170	0.50160	0.59520	0.0250*	
H19	0.74290	1.13120	0.55290	0.0240*	
H20	0.64380	1.13240	0.59070	0.0220*	
H21A	0.83040	0.52570	0.52050	0.0460*	
H21B	0.87210	0.57030	0.57390	0.0460*	
H21C	0.89700	0.65970	0.52100	0.0460*	

### Atomic displacement parameters (Å^2^)

**Table:**

	*U*^11^	*U*^22^	*U*^33^	*U*^12^	*U*^13^	*U*^23^
O1	0.0168 (4)	0.0180 (4)	0.0191 (4)	0.0025 (3)	0.0012 (3)	−0.0031 (3)
O2	0.0162 (4)	0.0278 (5)	0.0297 (5)	−0.0001 (4)	0.0079 (4)	−0.0048 (4)
N1	0.0184 (5)	0.0256 (6)	0.0243 (6)	0.0045 (4)	−0.0010 (4)	−0.0081 (5)
N2	0.0229 (5)	0.0203 (6)	0.0204 (5)	0.0018 (4)	0.0015 (4)	−0.0013 (4)
C1	0.0171 (6)	0.0171 (6)	0.0176 (6)	−0.0016 (4)	0.0052 (4)	−0.0018 (4)
C2	0.0169 (6)	0.0171 (6)	0.0179 (6)	−0.0010 (5)	0.0039 (4)	−0.0010 (5)
C3	0.0166 (6)	0.0169 (6)	0.0163 (6)	−0.0003 (4)	0.0023 (4)	0.0006 (5)
C4	0.0167 (6)	0.0177 (6)	0.0159 (6)	−0.0012 (5)	0.0055 (4)	0.0013 (5)
C5	0.0188 (6)	0.0204 (6)	0.0165 (6)	−0.0013 (5)	0.0049 (5)	0.0009 (5)
C6	0.0238 (6)	0.0262 (7)	0.0199 (6)	0.0044 (5)	0.0010 (5)	−0.0027 (5)
C7	0.0255 (7)	0.0398 (8)	0.0248 (7)	0.0073 (6)	−0.0039 (5)	−0.0037 (6)
C8	0.0283 (7)	0.0398 (8)	0.0213 (6)	−0.0005 (6)	−0.0041 (5)	−0.0086 (6)
C9	0.0268 (7)	0.0261 (7)	0.0210 (6)	−0.0020 (5)	0.0045 (5)	−0.0057 (5)
C10	0.0209 (6)	0.0199 (6)	0.0170 (6)	−0.0026 (5)	0.0063 (5)	−0.0003 (5)
C11	0.0231 (6)	0.0164 (6)	0.0180 (6)	−0.0017 (5)	0.0078 (5)	−0.0003 (5)
C12	0.0183 (6)	0.0179 (6)	0.0188 (6)	0.0010 (5)	0.0062 (5)	0.0025 (5)
C13	0.0170 (6)	0.0183 (6)	0.0143 (5)	−0.0022 (4)	0.0038 (4)	0.0015 (4)
C14	0.0156 (6)	0.0197 (6)	0.0160 (6)	−0.0003 (4)	0.0024 (4)	0.0019 (5)
C15	0.0149 (6)	0.0189 (6)	0.0146 (5)	−0.0004 (4)	−0.0001 (4)	−0.0024 (4)
C16	0.0199 (6)	0.0165 (6)	0.0213 (6)	0.0000 (5)	0.0015 (5)	0.0007 (5)
C17	0.0178 (6)	0.0194 (6)	0.0240 (6)	0.0046 (5)	0.0008 (5)	−0.0031 (5)
C18	0.0139 (5)	0.0257 (6)	0.0172 (6)	−0.0013 (5)	0.0019 (4)	−0.0053 (5)
C19	0.0213 (6)	0.0181 (6)	0.0203 (6)	−0.0025 (5)	0.0031 (5)	0.0001 (5)
C20	0.0185 (6)	0.0163 (6)	0.0192 (6)	0.0022 (4)	0.0016 (4)	−0.0016 (5)
C21	0.0197 (6)	0.0321 (8)	0.0410 (8)	0.0025 (5)	0.0089 (6)	−0.0137 (6)

### Geometric parameters (Å, º)

**Table:**

O1—C1	1.3625 (15)	C11—C12	1.3650 (18)
O1—C13	1.3871 (14)	C12—C13	1.4132 (17)
O2—C18	1.3740 (15)	C15—C20	1.3970 (17)
O2—C21	1.4338 (15)	C15—C16	1.3851 (17)
N1—C1	1.3438 (16)	C16—C17	1.3955 (18)
N2—C14	1.1538 (16)	C17—C18	1.3843 (18)
C1—C2	1.3607 (17)	C18—C19	1.3944 (17)
N1—H1B	0.900 (17)	C19—C20	1.3862 (17)
N1—H1A	0.896 (18)	C3—H3	1.0000
C2—C14	1.4184 (17)	C6—H6	0.9500
C2—C3	1.5166 (17)	C7—H7	0.9500
C3—C4	1.5149 (16)	C8—H8	0.9500
C3—C15	1.5311 (16)	C9—H9	0.9500
C4—C13	1.3733 (17)	C11—H11	0.9500
C4—C5	1.4326 (17)	C12—H12	0.9500
C5—C10	1.4307 (18)	C16—H16	0.9500
C5—C6	1.4197 (18)	C17—H17	0.9500
C6—C7	1.3716 (19)	C19—H19	0.9500
C7—C8	1.413 (2)	C20—H20	0.9500
C8—C9	1.367 (2)	C21—H21A	0.9800
C9—C10	1.4178 (18)	C21—H21B	0.9800
C10—C11	1.4194 (18)	C21—H21C	0.9800
			
C1—O1—C13	118.84 (9)	C15—C16—C17	121.65 (11)
C18—O2—C21	117.01 (10)	C16—C17—C18	119.19 (11)
O1—C1—N1	111.07 (10)	O2—C18—C19	116.12 (11)
O1—C1—C2	121.89 (11)	O2—C18—C17	123.96 (11)
N1—C1—C2	127.02 (12)	C17—C18—C19	119.92 (12)
H1A—N1—H1B	121.3 (15)	C18—C19—C20	120.29 (11)
C1—N1—H1A	118.7 (11)	C15—C20—C19	120.45 (11)
C1—N1—H1B	116.7 (11)	C2—C3—H3	107.00
C1—C2—C3	124.15 (11)	C4—C3—H3	107.00
C1—C2—C14	118.75 (11)	C15—C3—H3	107.00
C3—C2—C14	117.06 (10)	C5—C6—H6	119.00
C2—C3—C4	109.67 (10)	C7—C6—H6	119.00
C4—C3—C15	114.55 (10)	C6—C7—H7	120.00
C2—C3—C15	109.98 (9)	C8—C7—H7	120.00
C5—C4—C13	118.03 (10)	C7—C8—H8	120.00
C3—C4—C5	120.65 (10)	C9—C8—H8	120.00
C3—C4—C13	121.29 (10)	C8—C9—H9	119.00
C4—C5—C6	122.51 (11)	C10—C9—H9	119.00
C4—C5—C10	119.65 (11)	C10—C11—H11	119.00
C6—C5—C10	117.83 (11)	C12—C11—H11	119.00
C5—C6—C7	121.22 (12)	C11—C12—H12	120.00
C6—C7—C8	120.70 (13)	C13—C12—H12	120.00
C7—C8—C9	119.67 (13)	C15—C16—H16	119.00
C8—C9—C10	121.15 (12)	C17—C16—H16	119.00
C5—C10—C9	119.42 (11)	C16—C17—H17	120.00
C5—C10—C11	119.01 (11)	C18—C17—H17	120.00
C9—C10—C11	121.56 (11)	C18—C19—H19	120.00
C10—C11—C12	121.01 (11)	C20—C19—H19	120.00
C11—C12—C13	119.23 (11)	C15—C20—H20	120.00
C4—C13—C12	123.00 (11)	C19—C20—H20	120.00
O1—C13—C12	113.14 (10)	O2—C21—H21A	109.00
O1—C13—C4	123.86 (10)	O2—C21—H21B	109.00
N2—C14—C2	177.37 (12)	O2—C21—H21C	109.00
C3—C15—C16	119.06 (10)	H21A—C21—H21B	109.00
C3—C15—C20	122.33 (10)	H21A—C21—H21C	109.00
C16—C15—C20	118.49 (10)	H21B—C21—H21C	109.00
			
C13—O1—C1—N1	−178.67 (10)	C5—C4—C13—O1	−177.22 (10)
C13—O1—C1—C2	2.86 (17)	C5—C4—C13—C12	2.37 (18)
C1—O1—C13—C4	−0.19 (16)	C4—C5—C6—C7	−179.38 (13)
C1—O1—C13—C12	−179.81 (10)	C10—C5—C6—C7	−0.91 (19)
C21—O2—C18—C17	8.50 (17)	C4—C5—C10—C9	−179.71 (11)
C21—O2—C18—C19	−171.07 (11)	C4—C5—C10—C11	1.72 (18)
O1—C1—C2—C3	−6.34 (19)	C6—C5—C10—C9	1.78 (18)
O1—C1—C2—C14	175.95 (11)	C6—C5—C10—C11	−176.80 (12)
N1—C1—C2—C3	175.45 (12)	C5—C6—C7—C8	−0.7 (2)
N1—C1—C2—C14	−2.3 (2)	C6—C7—C8—C9	1.4 (2)
C1—C2—C3—C4	6.28 (16)	C7—C8—C9—C10	−0.5 (2)
C1—C2—C3—C15	−120.55 (13)	C8—C9—C10—C5	−1.10 (19)
C14—C2—C3—C4	−175.97 (10)	C8—C9—C10—C11	177.44 (13)
C14—C2—C3—C15	57.20 (14)	C5—C10—C11—C12	0.73 (19)
C2—C3—C4—C5	174.52 (11)	C9—C10—C11—C12	−177.82 (12)
C2—C3—C4—C13	−3.53 (15)	C10—C11—C12—C13	−1.63 (19)
C15—C3—C4—C5	−61.27 (14)	C11—C12—C13—O1	179.67 (11)
C15—C3—C4—C13	120.68 (12)	C11—C12—C13—C4	0.03 (19)
C2—C3—C15—C16	−93.24 (12)	C3—C15—C16—C17	176.32 (11)
C2—C3—C15—C20	82.78 (13)	C20—C15—C16—C17	0.14 (18)
C4—C3—C15—C16	142.72 (11)	C3—C15—C20—C19	−176.89 (10)
C4—C3—C15—C20	−41.26 (14)	C16—C15—C20—C19	−0.85 (17)
C3—C4—C5—C6	−2.87 (18)	C15—C16—C17—C18	0.34 (19)
C3—C4—C5—C10	178.69 (11)	C16—C17—C18—O2	−179.67 (11)
C13—C4—C5—C6	175.25 (12)	C16—C17—C18—C19	−0.12 (19)
C13—C4—C5—C10	−3.20 (17)	O2—C18—C19—C20	179.00 (11)
C3—C4—C13—O1	0.88 (17)	C17—C18—C19—C20	−0.59 (19)
C3—C4—C13—C12	−179.53 (11)	C18—C19—C20—C15	1.08 (18)

### Hydrogen-bond geometry (Å, º)

Cg2 is the centroid of the C4/C5/C10–C13 ring.

**Table:**

*D*—H···*A*	*D*—H	H···*A*	*D*···*A*	*D*—H···*A*
N1—H1*A*···N2^i^	0.896 (18)	2.125 (18)	3.0174 (15)	173.8 (16)
N1—H1*B*···O2^ii^	0.900 (17)	2.053 (17)	2.9509 (14)	175.5 (14)
C11—H11···*Cg*2^iii^	0.95	2.56	3.3913 (14)	147

Symmetry codes: (i) −*x*+1, −*y*+1, −*z*+1; (ii) *x*−1/2, −*y*+2, *z*; (iii) −*x*+1, *y*+1/2, −*z*+3/2.
